# Alterations and Correlations in Microbial Community and Metabolome Characteristics in Generalized Aggressive Periodontitis

**DOI:** 10.3389/fmicb.2020.573196

**Published:** 2020-11-30

**Authors:** Meng Shi, Yiping Wei, Yong Nie, Cui Wang, Fei Sun, Wenting Jiang, Wenjie Hu, Xiaolei Wu

**Affiliations:** ^1^Department of Periodontology, National Clinical Research Center for Oral Diseases, National Engineering Laboratory for Digital and Material Technology of Stomatology, Beijing Key Laboratory of Digital Stomatology, Peking University School and Hospital of Stomatology, Beijing, China; ^2^Laboratory of Environmental Microbiology, Department of Energy and Resources Engineering, College of Engineering, Peking University, Beijing, China

**Keywords:** aggressive periodontitis, subgingival microbiome, metabolome, high-throughput nucleotide sequencing, gas chromatography-mass spectrometry

## Abstract

This study aimed to characterize the microbial community and metabolic profiles in generalized aggressive periodontitis (AgP) using 16S ribosomal RNA (rRNA) gene high-throughput sequencing and gas chromatography-mass spectrometry (GC-MS). A total of 146 subgingival plaque samples and 50 gingival crevicular fluid (GCF) samples were collected from 24 patients with AgP and 10 periodontally healthy subjects (PH). Striking differences were observed in subgingival microbiome and GCF metabolomics between patients with AgP and PH, but not between samples with different probing depths (PDs). Metabolomics analysis combined with enrichment analysis showed that periodontitis significantly altered the concentration of compounds associated with biosynthesis of amino acids (e.g., alanine, leucine, isoleucine, and valine), galactose metabolism (e.g., myo-inositol, galactose, glucose, and hexitol), and pyrimidine metabolism (e.g., uracil, uridine, beta alanine, and thymine). Correlation analysis showed that the genera with significant difference between AgP and PH were usually significantly correlated with more metabolites, such as *Aggregatibacter*, *Rothia*, *Peptostreptococcaceae_[XI][G-5]*, and *Bacteroidaceae_[G-1]*. While glucose and oxoproline had the most significant correlations with microorganisms. Our results revealed distinct microbial communities and metabolic profiles between AgP and PH. The significant correlation between microbial taxa and metabolites suggested the possible mechanisms for periodontitis. Our results also provided effective approaches for detecting periodontal disease and managing periodontitis.

## Introduction

Periodontitis is a kind of inflammatory disease which causes destruction of the ligament and alveolar bone supporting the teeth, is the main cause of tooth loss in adults (Newman et al., 2011; Al-Harthi et al., 2013). There is considerable evidence that periodontitis is also a high risk factor for a variety of systemic diseases, including coronary heart disease (Tonetti et al., 2013), stroke (Sfyroeras et al., 2012), obesity (Suvan et al., 2011), atherosclerosis (Tonetti, 2009), diabetes (Lalla and Papapanou, 2011), premature birth, and low-weight newborn (Sanz et al., 2013). Aggressive periodontitis (AgP) is a rapidly progressive form of periodontal disease usually occurs in individuals younger than 35-year old that are different from chronic periodontitis (Armitage, 1999).

It is generally believed that plaque biofilm is the initiating factor of the onset of periodontitis, although the subsequent periodontal tissue destruction appears to be host-mediated. Due to technical limitations, for decades, the bacteriological etiology of periodontitis has been focusing on limited specific known species, such as *Porphyromonas gingivalis*, *Tannerella forsythia*, and *Treponema denticola* (Socransky et al., 1998). Implementation of next-generation sequencing technologies to understand microbial communities is changing our view of the human oral microbiome. Li et al. (2015) compared the similarity of bacterial profiles between AgP patients and their first-degree blood relatives with chronic periodontitis. Several studies reported microbial community shifts in AgP patients following non-surgical periodontal therapy (Campisciano et al., 2017; Han et al., 2017; Liu et al., 2018). Nevertheless, only a few studies applying high-throughput sequencing were conducted to assess the differences between subgingival bacterial compositions in patients with AgP and periodontally healthy subjects (PH; Shi et al., 2018; Schulz et al., 2019; Wei et al., 2019). These studies revealed that subgingival microbiome in AgP patients were dramatically altered, and periodontitis was not caused by one or several microorganisms, but the community behavior of all bacteria in the subgingival plaque. However, now microbiome research needs to study the metabolic activities of microorganisms and their role in the pathogenesis of diseases (Shaffer et al., 2017).

Metabolomic, as an emerging discipline, studies the metabolic pathways of biological systems, making that possible to find metabolite markers and providing a new entry point for exploring the pathogenesis of periodontal disease. Previous studies have shown that the molecular changes in saliva, serum, tongue coating, and gingival crevicular fluid (GCF) could indicate the development of chronic periodontitis (Barnes et al., 2009; Ozeki et al., 2016; Singh et al., 2017; Gawron et al., 2019). Elabdeen et al. (2013) also compared saliva, serum, and GCF in patients with AgP and healthy controls, in which the levels of n6- and n3- polyunsaturated fatty acids as well as various eicosanoids and docosanoids were significantly elevated in the AgP. Chen et al. (2018) successfully screened some differential metabolites in serum and GCF samples from patients with AgP, which implied that AgP might be related to oxidative stress, purine degradation, the metabolism of tyrosine and pyrimidine, and bacterial biochemistry.

At present, the commonly used methods for detecting metabolomics are nuclear magnetic resonance, gas chromatography-mass spectrometry (GC-MS), and liquid chromatography-mass spectrometry. GC-MS has high sensitivity and mature database, which is currently a suitable way for non-targeted metabolome detection.

Although several studies have revealed the distinctions in both microbial taxa and metabolites between the health and periodontitis, little is known about the relationship between microorganisms and metabolites. Integrating microbiological and metabonomic data helps to identify the physiological effects of microorganisms on host physiology by producing, modifying, or degrading bioactive metabolites. To the best of our knowledge, there are no reports combining omics approaches to explore the complex pathogenesis of AgP. Thus, in order to demonstrate the possible mechanisms for how the microorganisms and oral metabolites interact in periodontitis development, this study was designed to identify the microbial community and metabolic profiles in patients with generalized AgP and PH through a combination of the 16S ribosomal RNA (rRNA) gene high-throughput sequencing and GC-MS.

## Materials and Methods

### Study Population and Clinical Examination

The study protocol was approved by the Ethics Committee of the Peking University Health Science Center (approval number: PKUSSIRB-201631135). Written informed consent was obtained from all enrolled individuals in accordance with the Declaration of Helsinki.

From January 2017 to May 2018, 24 patients with generalized AgP were recruited from the Department of Periodontology, Peking University School and Hospital of Stomatology, and 10 PH subjects were selected as controls. The common inclusion criteria for all participants were that: (1) they agreed to join the research and signed an informed consent; (2) they were free of systemic disease and not pregnant or lactating; (3) they neither had not received any kind of periodontal treatment in the past half year, nor had taken antibiotics within the past 3 months; and (4) they were non-smoker. The clinical inclusion criteria for PH and the diagnostic criteria for AgP have been previously described in detail (Shi et al., 2018). The diagnostic criteria for AgP were based on the 1999 International Classification of Periodontal Diseases (Armitage, 1999).

Full-mouth clinical examinations were carried out by one practitioner (WH). Probing depth (PD), clinical attachment loss (CAL), plaque index (PLI), and bleeding index (BI) were recorded at six sites per tooth (Mazza et al., 1981). Calibration was described by Shi et al. (2018). Intraclass correlation coefficients for PD were 96% within 1 mm and AL were 90% within 1 mm.

### Sample Collection

Gingival crevicular fluid samples and subgingival plaque samples were collected 1 week after the full-mouth periodontal examination. All participants were requested to refrain from food for 8 h and oral hygiene for 12 h before sampling. Samples were obtained in the morning (around 8 AM to 9 AM) and put on ice immediately, and transported to the laboratory within 2 h and stored at −80°C before further processing.

Gingival crevicular fluid samples from mesiobuccal sites of molars with deep periodontal pockets (PD ≥ 7 mm) and sites with moderate pockets (4 mm ≤ PD ≤ 6 mm) were obtained of patients with AgP. For the healthy individuals, the mesial buccal sites of the first molars were the specified collection sites. After isolating the selected sampling area with cotton rolls and air drying gently, a Periopaper® (Oraflow Inc., NY, United States) filter strip was inserted gently and kept in the gingival sulcus for 30 s. Any filter strip visibly contaminated with blood or saliva was discarded. Samples from each participant were pooled into different site categories according to the PDs, stored in 1.5 ml EP tubes at −80°C until further use.

The subgingival plaque samples were collected after the collection of GCF from the same sites. The sampling method and preparation have been described elsewhere in detail (Shi et al., 2018).

### Microbial DNA Extraction and 16S rRNA Gene Sequencing

Genomic DNA was extracted from the subgingival plaque using the QIAamp DNA Mini Kit (QIAGEN Sciences, United States; Shi et al., 2018). The bacterial 16S rRNA V4 gene was analyzed to evaluate the bacterial composition and diversity using Illumina Hiseq (Novogene Bioinformatics Technology Co., Ltd.). PCR amplification of the V4 region of the bacterial 16S rRNA gene was performed using specific primers as described previously (Shi et al., 2018).

The sequencing process was described in previous studies (Shi et al., 2018). After filtered and trimmed, the raw reads were classified into operational taxonomic units (OTUs). Sequences with ≥97% similarity were assigned to the same OTUs. The taxonomy of each sequence was picked for each OTU and analyzed by RDP Classifier tool^1^ against the Human Oral Microbiome Database using a default confidence threshold of 0.7 (Dewhirst et al., 2010). The raw reads were deposited into the NCBI Sequence Read Archive database (Accession Number: SRP173111; SRP228020).

### Gingival Crevicular Fluid Sample Preparation

Six hundred microliters of methanol-water (methanol/water mix proportion: 3/1) was added to each GCF samples, and in order to prepare the internal standard, 20 μl of 2-chloro-l-phenylalanine (0.3 mg/ml) was added to EP tubes with the samples and dissolved in methanol. After vortexing for 30 s, an ultrasonic extraction of ice-cold water bath was conducted for 20 min. Subsequently, the samples were left at −20°C for 30 min. Then, the solution was centrifuged (10,000 rpm, 10 min) at 4°C. Four hundred microliters of supernatant in a glass vial was dried by centrifugal concentrator dryer, and 80 μl of 15 mg/ml methoxylamine hydrochloride in pyridine was subsequently added. The resultant mixture was vortexed vigorously for 2 min and incubated at 37°C for 90 min. Eighty microliters of BSTFA (with 1% TMCS) and 20 μl of n-Hexane were added into the mixture, which was derivatized at 70°C for 60 min prior to injection. Eventually, the solutions were incubated for 30 min at room temperature.

### Gas Chromatography-Mass Spectrometry Analysis

All GCF samples were analyzed together in the same batch, and laboratory personnel were not aware of sample grouping. A GC-MS system (7890A-5975C), along with a chromatographic column (HP-5MS; 30 m × 0.25 mm × 0.25 μm, Agilent J & W Scientific, Folsom, CA, United States), was used for the separation of derivatives. Under a controlled injector temperature (260°C), the splitless injection was performed with the carrier gas helium (1 ml/min) at an injection volume of 1 μl. The initial temperature was 60°C, and the speed was increased to 310°C at 8°C/min for 6 min.

The temperature of the MS quadrupole was set to 150°C and the temperature of the ion source was 230°C. Collision energy of 69 eV was applied. The mass scan ranged from m/z 50 to 600 in a full-scan mode. Continuous sample analysis was performed in random order to avoid the effects of instrument signal fluctuations. Quality control (QC) samples were prepared by mixing the extracts of all samples of equal volume. Each QC sample had the same volume and was processed in the same way as the analytical sample. During the analysis, between every 10 analyses, a QC sample was inserted into the sample to examine the repeatability of the entire analysis process.

### Bioinformatic Analysis, Statistical Analysis, and Visualization

16S rRNA sequence data analyses were performed using QIIME software package (Quantitative Insights Into Microbial Ecology), and in-house Perl scripts were used to analyze alpha- (within samples) and beta- (among samples) diversity (Caporaso et al., 2010). Two metrics were calculated for the evaluation of alpha diversity after rarified the OTU table: abundance-based coverage estimator (ACE) estimates the species abundance; and the diversity of the sample microbiota was estimated by the Shannon index. Normality tests for each group of data were conducted. The Student’s *t*-test was used to compare significant differences of the alpha diversity indexes between the different groups (*p* < 0.05). A principal coordinates analysis (PCoA) based on unweighted UniFrac distance metrics was conducted on the OTU level to evaluate the phylogenetic structures of microbial community structure among various groups. The taxonomy compositions and abundances were visualized by GraphPad PRISM® software (version 4.0). When comparing the taxa in subgingival plaque between PH and AgP, the Mann-Whitney test was used. The Mann-Whitney test and Student’s *t*-test were performed using SPSS 20.0.

The raw data of GC-MS (D format) were converted to the standard format (CDF format) by ChemStation analysis software (version E.02.02.1431, Agilent, CA, United States), and then imported into Chroma TOF software (version 4.34, LECO, St Joseph) for preprocessing, including peak extraction, denoising, deconvolution, etc. The NIST database was used to characterize the metabolites, and the data were normalized by internal standards and pseudo-positive peaks, such as peaks caused by noise, column bleed, and the BSTFA derivatization procedure, were removed, eventually a three-dimensional data matrix was derived in CSV format (raw data matrix). The three-dimensional matrix included information such as the name of each substance peak, retention time, mass-to-nuclear ratio, and mass spectrometry response intensity (peak area). The resulting data matrix was imported into the SIMCA-P + 14.0 software package (Umetrics, Umeå, Sweden). Unsupervised principal component analysis (PCA) was first used to observe the overall distribution between samples and the stability of the entire analytical process. A supervised (orthogonal) partial least squares analysis [(O) PLS-DA] was then used to find differential metabolites between the groups. In order to prevent the model from overfitting, the quality of the model was examined by using a default seven-round interactive verification, and the (O) PLS-DA models were also validated by a permutation analysis (200 times). In the (O) PLS-DA analysis, variable influence on projection (VIP) value greater than one and values of *p* (two-tailed Student’s *t*-test) less than 0.05 were considered to be a different variable. Metabolites were then identified by searching in a self-built database of the Majorbio I-Sanger Cloud Platform,^2^ which integrating the data of Human Metabolome Database, kyoto encyclopedia of genes and genomes (KEGG) Compound Database, and Lipid Maps Structure Database.

The correlative relationship among microbial communities, metabolites, and clinical indices were conducted through Spearman’s correlation analysis, and visualized as heatmap.

## Results

### Microbial Profiles of Subgingival Plaque Samples

A total of 34 subjects, including 24 patients with AgP and 10 PH, were enrolled in this study. Demographic and clinical characteristics are shown in Table 1. As expected, PLI, PD, AL, and BI values were significant higher in AgP than PH (*p* < 0.05).

**Table 1:** Demographic and clinical characteristics of the study population.

	AgP (*n* = 24)	PH (*n* = 10)
Age	29.4 ± 4.2	26.5 ± 1.7
Gender		
Male	9	4
Female	15	6
Sampled sites		
PD (mm)	6.0 ± 1.7	2.7 ± 0.5
BI	3.8 ± 0.3	0.8 ± 0.6
CAL (mm)	5.3 ± 2.3	0
PLI	2.04 ± 0.58	0.61 ± 0.65

Values are means ± SDs. PD, probing depth; CAL, clinical attachment loss; BI, bleeding index; and PLI, plaque index.

Two groups were identified among samples from patients with AgP. The sample groups are designated as AgP_GD (samples from deep periodontal pockets) and AgP_GM (samples from moderate periodontal pockets). In this study, a total of 146 samples of subgingival plaque were collected, including 44 samples in AgP_GD, 81 samples in AgP_GM, and 21 samples in PH.

Sequences were clustered to 1,164 OTUs. A total of 12 phyla, 31 classes, 54 orders, 101 families, and 190 genera were detected. Rarefaction curves showed a diminishing rate of new OTU identification as the number of reads per sample increased, implying that the sampling depth was adequate for evaluating dominant members of the subgingival plaque’s bacterial community.

**Figure 1:**
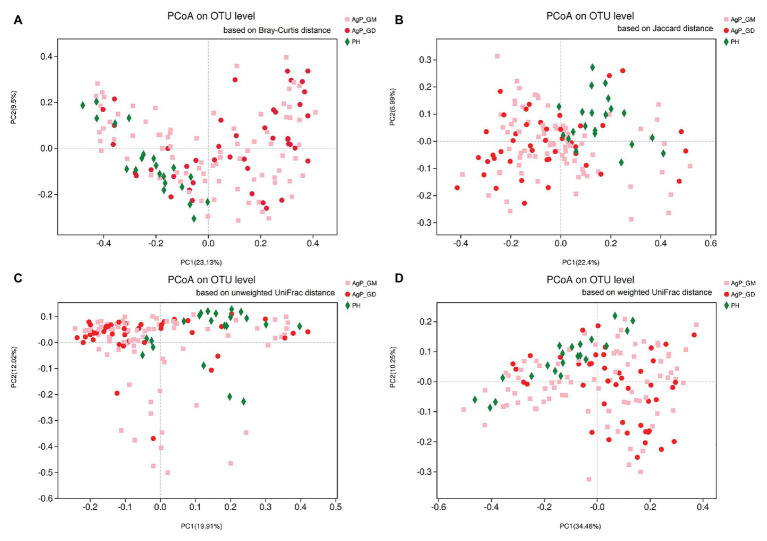
Beta-diversity of subgingival plaque samples in three groups. Principal coordinates analysis (PCoA) of Bray-Curtis distance **(A)**, Jaccard distance **(B)**, unweighted UniFrac distance **(C)**, and weighted UniFrac distance **(D)** were performed based on the operational taxonomic unit (OTU) abundances.

First, we analyzed the distribution of microbiota among three groups by PCoA, which were conducted based on the OTU abundances using several different matrix distances in order to consider both species richness and evolutionary distance between species. The results showed that the subgingival plaques of periodontitis patients and healthy subjects formed different clusters and could be separated, while AgP_GD and AgP_GM samples clustered together in every matrix distance which could not be distinguished from each other (Figure 1). Three nonparametric statistical methods, ANOSIM (analysis of similarity), Adonis (permutational multivariate analysis of variance), and MRPP (multi-response permutation procedure) were used to further confirm that there was no significant difference between group AgP_GM and AgP_GD (*p* > 0.05; Table 2). Therefore, we combined these two groups into the AgP group for further analysis.

**Table 2:** Dissimilarity comparison of microbial community and metabolome among groups using three nonparametric statistical methods (*p* value).

Groups		ANOSIM	adonis	MRPP
Microbial community of subgingival plaque	AgP vs. PH	0.366	0.001^***^	0.001^***^
	AgP_GM vs. PH	0.579	0.001^***^	0.001^***^
	AgP_GD vs. PH	0.001^***^	0.001^***^	0.001^***^
	AgP_GM vs. AgP_GD	0.333	0.071	0.08
Metabolome of gingival crevicular fluid (GCF)	AgP vs. PH	0.001^***^	0.001^***^	0.001^***^
	AgP_GM vs. PH	0.001^***^	0.001^***^	0.001^***^
	AgP_GD vs. PH	0.001^***^	0.001^***^	0.001^***^
	AgP_GM vs. AgP_GD	0.545	0.726	0.704

adonis, non-parametric multivariate analysis of variance (MANOVA) with the adonis function; ANOSIM, analysis of similarity; and MRPP, multi-response permutation procedure.

*** *p* ≤ 0.001.

**Figure 2:**
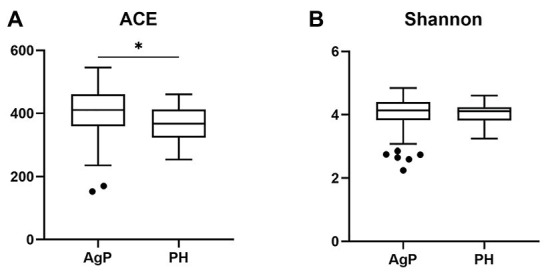
Alpha-diversity of bacterial communities of aggressive periodontitis (AgP) and periodontally healthy subjects (PH). Microbiota alpha-diversity as calculated by abundance-based coverage estimator (ACE) index **(A)** and Shannon index **(B)** of subgingival plaque samples in PH and AgP group. The error bars indicate mean with SE. ^*^p < 0.05.

Compared with the PH samples, the ACE index of the subgingival microbial community significantly increased in patients with AgP. A slightly higher Shannon index was found in AgP group, but no statistical significance was detected (*p* > 0.05; Figure 2).

**Figure 3:**
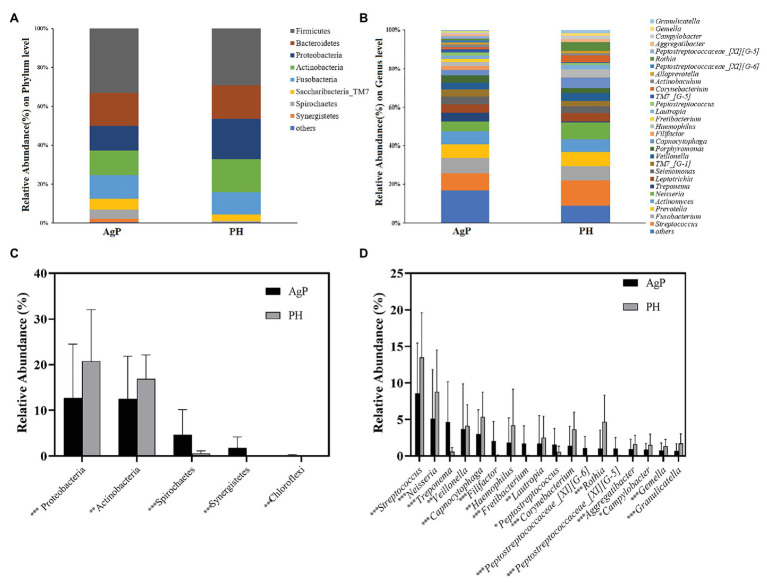
Bacterial compositions of AgP and PH. Relative abundance of bacterial composition at the phylum level **(A)** and genus level **(B)** of subgingival plaque samples in health group (shown as PH) and AgP group (shown as AgP). Comparisons of microbiota that presented significantly different contents in subgingival plaque of AgP and PH at phylum level **(C)** and genus level **(D)**. ^*^p < 0.05; ^**^p < 0.01; and ^***^p < 0.001.

In the AgP and PH groups, the five most abundant phyla were *Firmicutes*, *Bacteroidetes*, *Proteobacteria*, *Actinobacteria*, and *Fusobacteria*, which represented more than 85% of the total sequences (Figure 3A). At the genus level, the five most abundant in subgingival plaque samples were *Streptococcus*, *Fusobacterium*, *Prevotella*, *Actinomyces*, and *Neisseria*, *Leptotrichia* represented more than 50% of the total sequences among the 190 genera (Figure 3B).

Each of the 12 phyla and 190 genera were compared between samples from periodontitis and healthy subjects. The abundance of *Proteobacteria* and *Actinobacteria* was significantly higher in PH, but other phyla such as Spirochaetes, Synergistetes, and Chloroflexi increased significantly in AgP (Figure 3C). At the genus level, there were 60 genera distributed differently between two groups. In genera with relative abundance greater than 1%, *Filifactor*, *Fretibacterium*, *Peptostreptococcaceae_[XI][G-5]*, *Peptostreptococcaceae_[XI][G-6]*, *Peptostreptococcus*, and *Treponema* were significantly higher in AgP group, while *Aggregatibacter*, *Campylobacter*, *Capnocytophaga*, *Corynebacterium*, *Gemella*, *Granulicatella*, *Haemophilus*, *Lautropia*, *Neisseria*, *Rothia*, *Streptococcus*, and *Veillonella* showed higher abundances in healthy controls (Figure 3D).

### Metabolic Profiles of Gingival Crevicular Fluid Samples

As for metabolites analysis of GCF, after pooling each patient’s samples from the same category of periodontal pocket depth, metabolomics analysis was performed on 50 samples, including 19 samples in AgP_GD, 21 samples in AgP_GM and 10 samples in PH.

The total ion current chromatograms of the GCF samples revealed that some spectrum peaks exist significant differences between groups. After removing the internal standards and pseudo-positive peaks and combining the peaks from the same metabolite, a total of 134 qualitative metabolites were used in the subsequent analysis.

The dissimilarity tests, including ANOSIM, Adonis, and MRPP, indicated no significant difference in metabolites between group AgP_GM and AgP_GD either (Table 2). Thus, we combined these two groups into the AgP group for further analysis.

**Figure 4:**
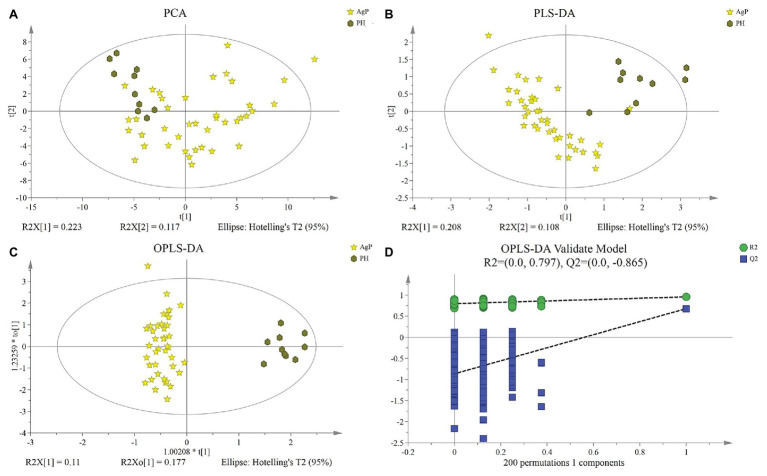
Differences of metabolic profiles between AgP and PH. Principal component analysis (PCA) analysis **(A)**, partial least-squares discriminant analysis (PLS-DA) analysis **(B)**, (orthogonal) PLS-DA [(O) PLS-DA] analysis **(C)** of the metabolic profiles of gingival crevicular fluid (GCF) samples from AgP and PH, and response permutation testing (RPT) of OPLS-DA **(D)**. GCF, gingival crevicular fluid; AgP, aggressive periodontitis; PCA, principal components analysis; PLS-DA, partial least-squares discriminant analysis; OPLS-DA, orthogonal partial least-squares discriminant analysis; and RPT, response permutation testing.

Multivariate analysis between different groups was performed in PCA, PLS-DA, and OPLS-DA. A trend of divergence was shown in GCF samples between the PH and the AgP in both PD categories by the plot of PCA scores. The OPLS-DA model for AgP vs. PH demonstrated satisfactory modeling and predictive abilities. The OPLS-DA model demonstrated a distinct separation between the metabolite profiles of these groups (Figure 4).

**Figure 5:**
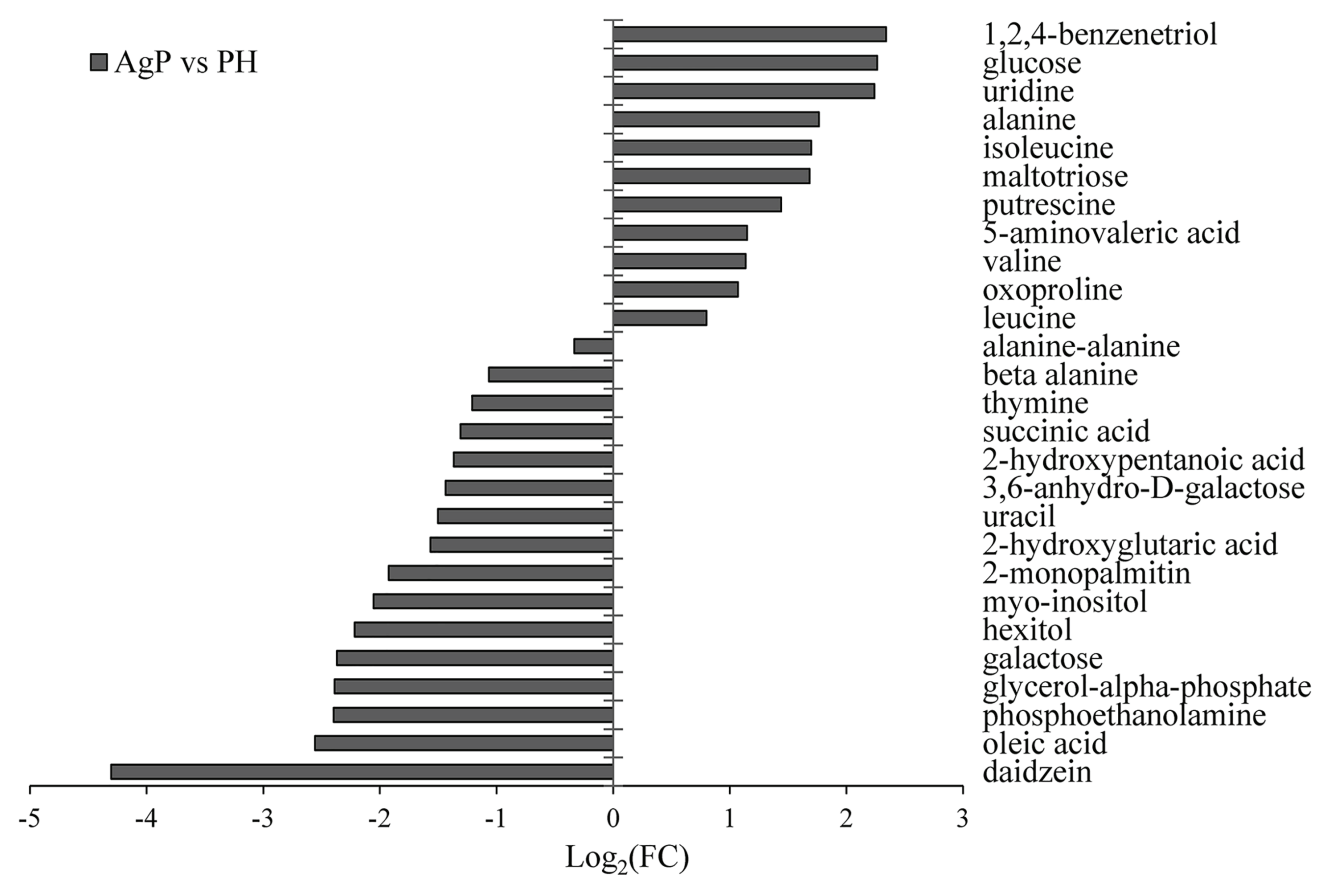
Significantly different metabolites in AgP compared with PH. Values of log2 (FC) resulting from the comparison between AgP and PH. Log2 (FC) with a positive value indicates a relatively higher concentration present in AgP, while a negative value means a relatively higher concentration in the PH.

A total of 103 GCF metabolites were identified using GC-MS analysis. The differences in GCF samples between groups were distinguished by VIP values and two-tailed Student’s *t*-tests. As the results shown that 27 potential biomarkers were selected between groups AgP and PH, based on *p* < 0.05 and VIP value > 1. These compounds were listed in Figure 5. Among these compounds, the levels of glucose, uridine, alanine, isoleucine, maltotriose, putrescine, 5-aminovaleric acid, valine, oxoproline, and leucine were significantly higher in AgP, while galactose, hexitol, myo-inositol, 3,6-anhydro-D-galactose, uracil, thymine, and beta alanine were significantly higher in PH.

### Pathways and Enrichment

**Figure 6:**
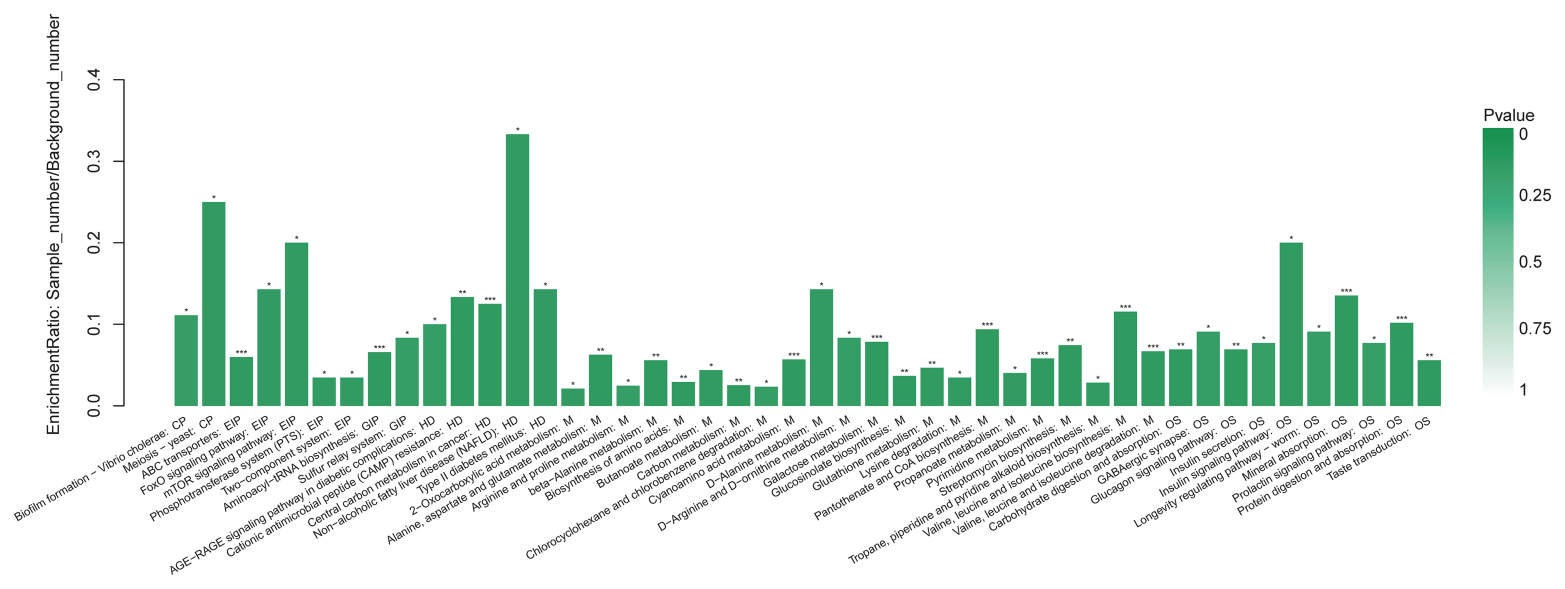
Functional enrichment analysis of GCF metabolites with significant difference between AgP and PH. Each column in the figure represents a pathway; the abscissa text represents the name of the pathway. The height of the column represents the Enrichment rate. ^*^p < 0.05, ^**^p < 0.01, and ^***^p < 0.001.

We annotated the differential metabolites and classified them according to their participating pathways or functioning through the KEGG database annotation. Hierarchical classification was conducted according to the KEGG metabolic pathways with different metabolites involved. Then, we conducted enrichment analysis of differential metabolite KEGG pathway (Figure 6). The method of enrichment analysis is usually to analyze whether a group of metabolites have been present at a certain functional node. The enrichment analysis can identify the biological processes most related to biological phenomena.

When compared AgP and PH, there were several pathways containing more kinds of differential metabolites, including protein digestion and absorption, biosynthesis of amino acids (including alanine, leucine, isoleucine, and valine), galactose metabolism (including myo-inositol, galactose, glucose, and hexitol), valine, leucine, and isoleucine biosynthesis (including leucine, valine, and isoleucine), 2-Oxocarboxylic acid metabolism (including leucine, valine, and isoleucine), and pyrimidine metabolism (including uracil, uridine, beta alanine, and thymine).

### Correlation Between Microbial Community and Metabolome Characteristics

To better understand the relationships among the subgingival plaque microbiota, GCF metabolites, we performed a correlation analysis of the microbial diversity at the phylum and genus level with the differential metabolites and constructed the related network [(|correlation r|) > 0.25, *p* < 0.05].

**Figure 7:**
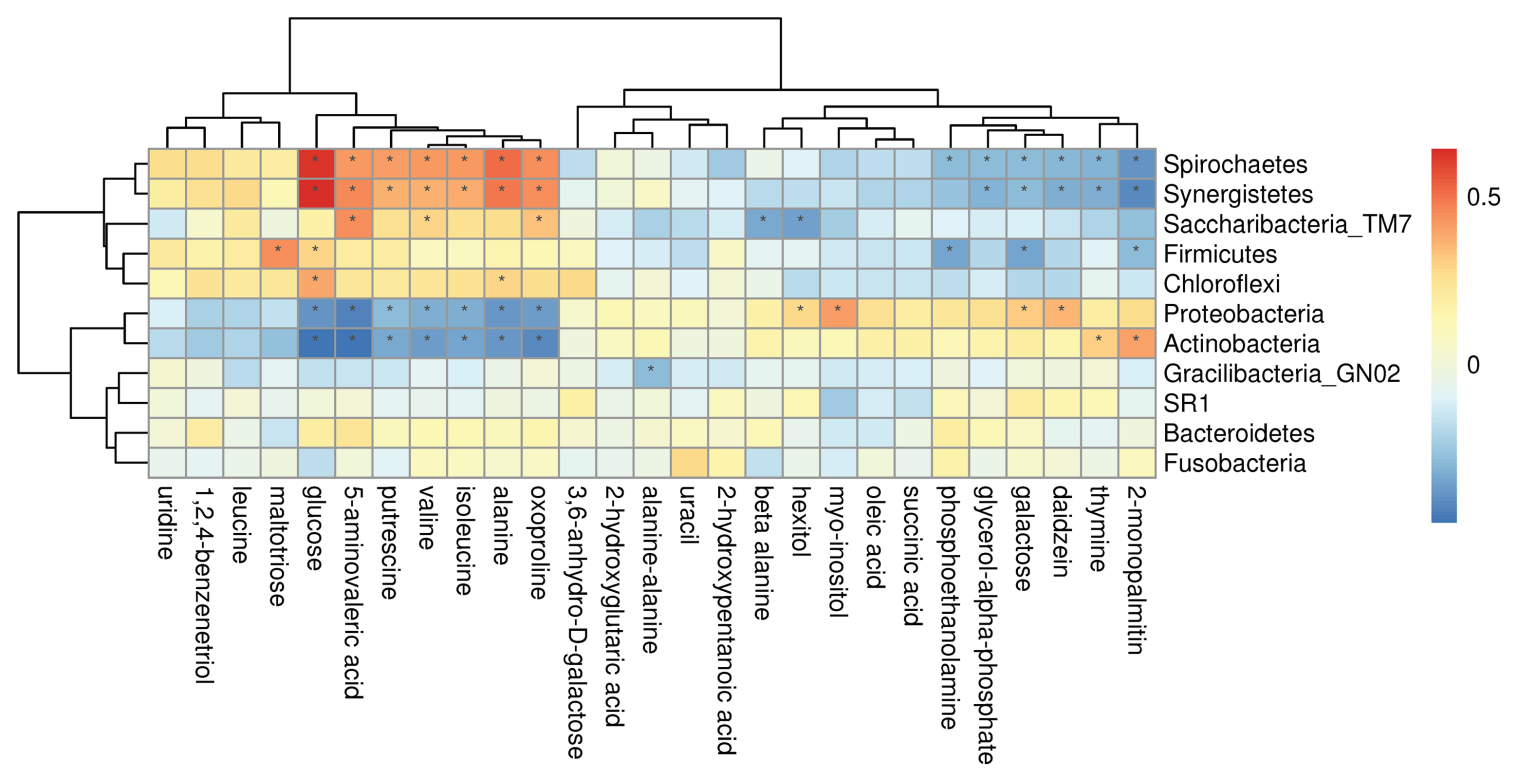
Correlations between subgingival plaque bacteria (phylum level) and metabolites in GCF. Each row in the graph represents a metabolite, each column represents a phylum, and each lattice represents a correlation coefficient between a component and a metabolite. Red represents a positive correlation, while blue represents a negative correlation. ^*^Significant correlation between the phyla and metabolites (*p* < 0.05).

**Figure 8:**
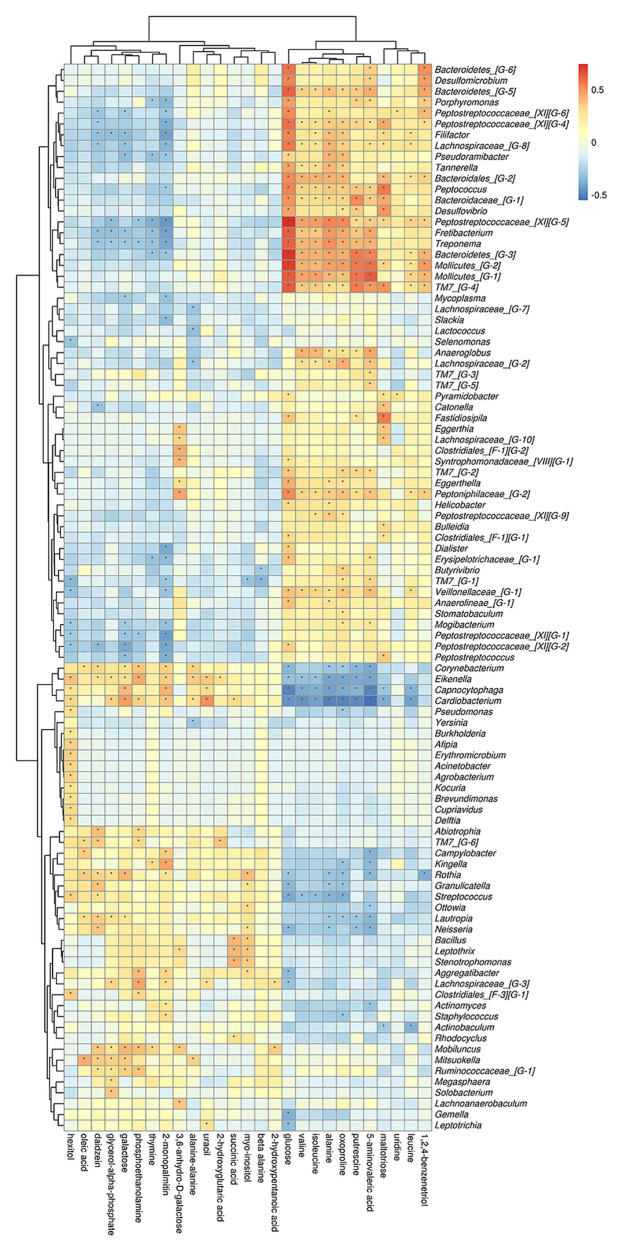
Correlations between subgingival plaque bacteria (genus level) and metabolites in GCF. Each row in the graph represents a metabolite, each column represents a genus, and each lattice represents a correlation coefficient between a component and a metabolite. Red represents a positive correlation, while blue represents a negative correlation. ^*^Significant correlation between the genera and metabolites (*p* < 0.05).

On phylum level, by comparing AgP and PH groups, we found that Spirochaetes, Synergistetes, and *Proteobacteria* were the most closely related to the metabolites, such as glucose, putrescine, and alanine. Saccharibacteria_TM7, Synergistetes’s relationship with metabolites always had been the opposite relationship with *Proteobacteria* (Figure 7). On genus level, *Cardiobacterium*, *Eikenella*, *Peptostreptococcaceae_[XI][G-5]*, *Capnocytophaga*, *Treponema*, and *Rothia* were the genera with the most correlations with differential metabolites, which had significant correlations with 17, 16, 14, 13, and 13 metabolites, respectively (Figure 8).

Analysis from the perspective of metabolites showed that glucose and oxoproline had the most significant correlations with microorganisms, which were significantly correlated with 46 and 39 different bacteria genera, respectively. Secondly, 5-aminovaleric acid, alanine, 2-monopalmitin were significantly correlated with 36, 34, and 32 different genera.

### Correlation Between the Microbiota, Metabolites, and Periodontal Clinical Indices

By using Spearman’s correlation analysis, the correlations between microbiota, metabolites and periodontal clinical data were reviewed.

**Figure 9:**
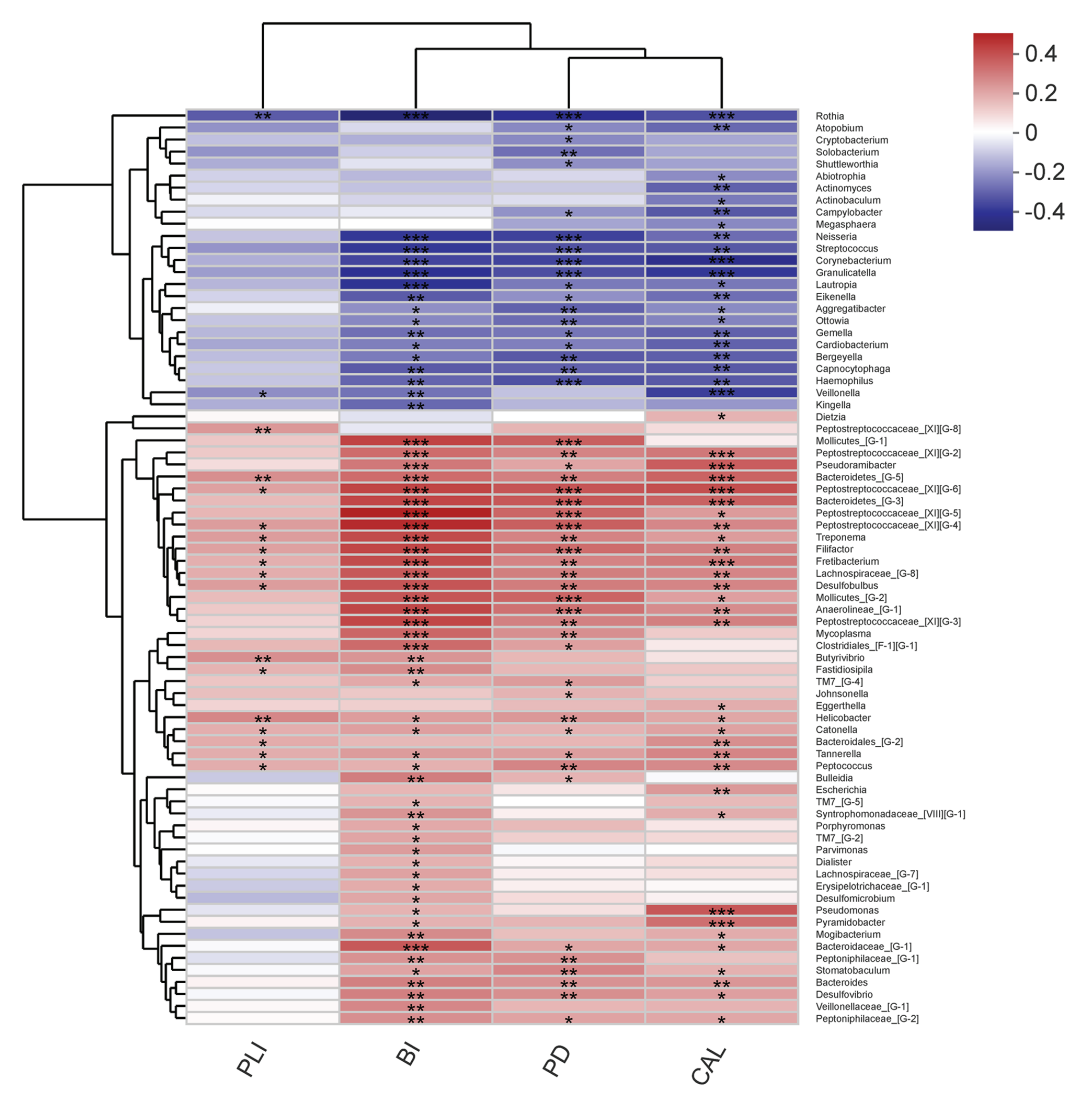
Correlations among the microbiome and periodontal clinical indices shows as a heatmap (only genera correlating with at least one clinical index with *p* < 0.05 are shown). Significant correlation between the genera and clinical indices (^*^p < 0.05, ^**^p < 0.01, and ^***^p < 0.001).

The relationship between microbiota and periodontal clinical indices was showed in heatmap (Figure 9). Over 70 genera had significant correlation with at least one clinical index of periodontitis. Some well-known periodontitis-associated genera were positively correlated with clinical indicators, such as *Treponema*, *Filifactor*, *Tannerella*, and *Peptostreptococcaceae_[XI][G-6]*. Other genera, such as *Rothia*, *Neisseria*, and *Veillonella* had negative relationship with these indicators of periodontitis.

**Figure 10:**
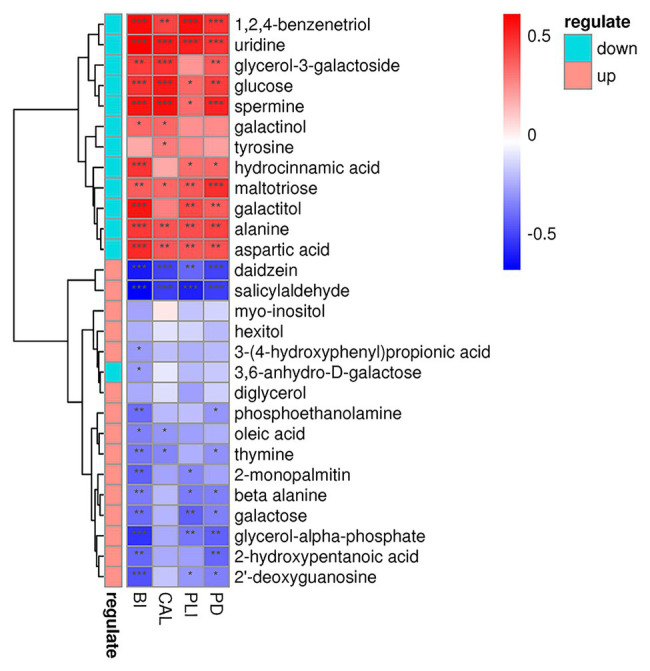
Correlations among the metabolites and periodontal clinical indices shows as a heatmap. Metabolites in green and red classes represent downregulated and upregulated in the periodontitis group, respectively. Significant correlation between the metabolites and clinical indices (^*^p < 0.05, ^**^p < 0.01, and ^***^p < 0.001).

As for metabolites, we found that glucose, alanine, and aspartic acid had positive relationship with increased clinical indices, while daidzein, galactose, and oleic acid occur the polar tendency (Figure 10). These results indicated us that, metabolites with elevated concentrations in patient’s GCF, were often positively correlated with the increased clinical indicators.

## Discussion

In this study, we used 16S rRNA gene sequencing and GC-MS to evaluate and compare the differences of the characteristics of subgingival microbiome and their metabolites between patients with generalized AgP and PH. In 2018, a new classification system of periodontal diseases was introduced (Tonetti et al., 2018). According to the new classification, the included patients with AgP in this study fit to the generalized stage III to IV, grade C periodontitis.

The results showed greater microbial richness in patients with AgP compared to healthy controls, but no significant differences in diversity between the two groups. This result is in agreement with previous study (Shi et al., 2018; Schulz et al., 2019). However, the outcomes from different studies mainly based on patients with chronic periodontitis are not consistent (Griffen et al., 2012; Abusleme et al., 2013; Ai et al., 2017; Wei et al., 2019). This kind of difference brings up another hint that besides the microbial challenge, the host defenses and the environment factors also need to be considered.

Differences in bacterial community composition between AgP and PH were found in the present study. Bacteria of the phyla Spirochaetes and Synergistetes were increased in AgP group compared with PH, which in accordance with previous results (Schulz et al., 2019; Wei et al., 2019). Schulz et al. (2019) identified the 10 biomarkers for AgP, including *Porphyromonas gingivalis*, *Treponema denticola*, *Tannerella forsythia*, and *Filifactor alocis*, using linear discriminant analysis effect size. This confirms our findings since genus levels of these species are significantly increased in AgP group shown in our study.

There has been little research comparing individual subgingival plaque samples from sites with different PDs (Ge et al., 2013; Pérez-Chaparro et al., 2018). Regarding to the study of Pérez-Chaparro et al. (2018) moderate (PD 4–6 mm) and deep (PD ≥ 7 mm) sites in chronic periodontitis subjects did not show major dissimilarities. In line with our results, we found the lack of marked differences in microbiome between AgP_GD and AgP_GM. Our results were consistent with the previous view that any pocket, regardless of its depth, might be a reservoir of periodontal pathogens which probably caused by the common features of periodontal pockets ≥4 mm, including redox potential, temperature, availability, and concentration of bacterial nutrients (Pérez-Chaparro et al., 2018). 16S rRNA gene sequencing by using a relatively quantitative method more reflects the structure of the dental plaques, and cannot fully reflect the actual situation of community biomass in the sample. Moreover, previous study had shown that in the sites of periodontitis, the clinical evidence of increased inflammation was often not related to the distinct microbiome, but more likely to correspond to higher microbial load (Abusleme et al., 2013). This might be the reason why there was no significant difference in the bacterial community structure between the sites with different periodontal PD.

Metabolome data revealed that periodontal disease altered the concentrations of metabolites in GCF which indicated that the metabolism might be linked with the periodontal microbiota activities. There were significant differences in the composition of metabolites in GCF between the AgP and the PH. Meanwhile, there was no significant difference between AgP_GD and AgP_GM, which was consistent with the results of the microbial diversity of subgingival plaque. Furthermore, the enrichment analysis showed that metabolic pathways, such as amino acid biosynthesis, pyrimidine metabolism, and galactose metabolism were significantly affected.

During the development of periodontitis, the levels of amino acid metabolites change, and some of these metabolites are produced by the degradation of dietary proteins by microorganisms in subgingival plaque (Duran-Pinedo and Frias-Lopez, 2015). Syrjänen et al. (1990) found that the levels of aspartate, threonine, serine, alanine, isoleucine, leucine, tyrosine, and ornithine in GCF of periodontitis patients were different from those of periodontal healthy controls. Our finding was partially consistent with the above studies, only the types and trends of some amino acids were not exactly the same. The reason for this inconsistency might be based on different testing methods. In addition, there were many factors affecting the changes of amino acids, and the reaction pathways involved are numerous, intricate, and overlapping. According to the correlative analysis of microbiome and metabolites in our research, aspartic acid, alanine, serine, isoleucine, and leucine were related to several genera. Most of them were also the genera whose proportion presented significant differences between PH and AgP, such as *Aggregatibacter*, *Anaeroglobus*, *Veillonellaceae_[G-1]*, *Peptostreptococcaceae_[XI][G-5]*, and *Bacteroidaceae_[G-1]*.

Beta alanine, which is proposed as an intermediate in the formation of acrylamide and acetonitrile or as a direct precursor of poly-beta-alanine, was normally metabolized into acetic acid. Acetic acid had been reported as a potential biomarker which had a positive relationship with AgP and might indicated the composition of subgingival plaque in AgP (Lu et al., 2013). In our study, although acetic acid did not show significant difference between groups, the levels of beta alanine were decreased in AgP when compared with PH. This phenomenon might suggest that beta alanine was also a biomarker for AgP.

Putrescine is a type of polyamine metabolism which often involved in maintaining tissue health and speeding up tissue repair. Previous studies have suggested that the increased concentration of putrescine might be involved in the process of recovery from inflammation of gingiva (Ishida et al., 1983). In this study, putrescine levels were significantly higher in the AgP, which was in accordance with Barnes et al. (2009). Study had shown that a significant increase in putrescine levels caused an increase in cell mitosis and might help repair cellular functions that connect damaged epithelial cells by stimulating specific proteins (Atkins et al., 1975). According to the correlation analysis between the diversity of subgingival plaque flora and metabolites of GCF, this study found that putrescine levels were positively correlated with *Bacteroidaceae_[G-1]* and *Peptostreptococcaceae_[XI][G-5]*, and negatively correlated with *Staphylococcus*, *Mobiluncus*, *Streptococcus*, and *Aggregatibacter*, which implied the source of putrescine might come from microorganisms of subgingival plaque. Still, more studies will need for exploring the exact mechanism between putrescine and periodontitis.

It is noteworthy that we found alterations in the concentrations of several metabolites associated with pyrimidine metabolism. Previous studied showed that pathogens could manipulate the pyrimidine metabolism of the host and create the suitable environment for them (Garavito et al., 2015). In this study, the levels of beta alanine and thymine decreased and the level of uridine increased in the AgP group, suggesting that pathogenic bacteria associated with periodontitis may alter the metabolic activity of their infected host through pyrimidine metabolism, thereby affecting the health of periodontal tissues. Although our result was consistent with the finding of Chen et al. (2018), the kinds and trends of metabolites involved in this pathway were not completely the same. This finding suggested that monitoring changes in a pathway might be more indicative of periodontal health than one or some metabolites.

It has been reported that most periodontitis-related bacteria could utilize amino acids and sugars as their energy and carbon substrates (Duran-Pinedo and Frias-Lopez, 2015). In previous studies, the concentration of ribulose-5-phosphate and glucose-1-phosphate increased in patients with AgP, and the level of fructose-5-phosphate was decreased in patient with chronic periodontitis when compared with healthy individuals. In our research, the levels of galactose, myo-inositol, and hexitol were decreased, while glucose increased in AgP group when compared with PH group. We found that these compounds above were all in the pathway of galactose metabolism. This result stated that periodontitis not only affected the periodontal tissue, but also coincides with previous studies on the bidirectional relationship between periodontitis and diabetes: on the one hand, diabetes was a risk factor for periodontitis; on the other hand, periodontitis had a negative impact on the metabolic control of diabetes. It had been recognized by the international medical community that periodontitis was the “sixth complication” of diabetes. However, the pathways of interaction and correlation between periodontitis and diabetes have not been fully elucidated so far. The results of this part of the study found that with the progress of periodontitis, galactose metabolism in GCF was active, leading to the upregulation of glucose levels in periodontal pockets, which we speculated may be one of the ways of interaction between periodontitis and diabetes. There was no report on the role of galactose metabolism in periodontitis, and our speculation still needed further study to confirm.

Combining metabolome and microbiome data has resulted in some exciting findings in health and disease (Zhao et al., 2018). Herein, we observed the significant correlation between subgingival microbiota genera and metabolites of GCF using correlation analysis. The genera with significant difference between AgP and PH groups were usually significantly correlated with more metabolites, such as *Aggregatibacter*, *Rothia*, *Peptostreptococcaceae_[XI][G-5]*, and *Bacteroidaceae_[G-1]*. Genera with the same change trend between AgP and PH group tended to have the similar correlation with some certain metabolites, positively or negatively. Besides some genera that had no significant difference between groups also showed significant correlation with some metabolites, such as *Actinobaculum*, *Lachnoanaerobaculum*, and *Olsenella*.

Furthermore, the results of correlation analysis suggested that the clinical health status of periodontal tissues was significantly correlated with both microorganisms and metabolites. As shown in our results, the genera with increased relative abundance in patients tended to be significantly positively correlated with increased clinical indicators, while genera with increased relative abundance in health showed an opposite relationship. This kind of relationship also existed in the relationship between metabolites and clinical indicators. However, due to the limitations of sequencing depth and lack of relevant literature, further investigation was required to clarify more detailed relationship.

In conclusion, striking differences were observed in subgingival microbiome and GCF metabolomics between patients with AgP and PH, but not between AgP_GD and AgP_GM. Correlation analysis of the relationship of microorganisms with metabolite features provides us with a comprehensive understanding of the composition and function of microbial communities. A longitudinal study is needed to verify these potential biomarkers. The current study does have some limitations. First, the sample size was small. Second, the relative abundance of the detected taxa was computed in each site. Nonetheless, this study applying multi-omics approaches suggested that these microbial and metabolic profiles have great potential in detecting AgP and helping to understand its underlying mechanisms.

## Data Availability Statement

The datasets presented in this study can be found in online repositories. The names of the repository/repositories and accession number(s) can be found in the article/supplementary material.

## Ethics Statement

The studies involving human participants were reviewed and approved by Ethics Committee of the Peking University Health Science Center (approval number: PKUSSIRB-201631135). The patients/participants provided their written informed consent to participate in this study.

## Author Contributions

WH and XW: conception and design. YW, MS, FS, and WJ: recruitment of patients and collection of oral samples. MS, YW, and YN: analysis and interpretation of data. YW and MS: manuscript preparation. MS, YW, YN, WH, and XW: manuscript revisions. All authors contributed to the article and approved the submitted version.

### Conflict of Interest

The authors declare that the research was conducted in the absence of any commercial or financial relationships that could be construed as a potential conflict of interest.

## Footnotes

^1^http://rdp.cme.msu.edu/

^2^https://www.i-sanger.com
